# Combinational Regularity Analysis (CORA) — a new method for uncovering complex causation in medical and health research

**DOI:** 10.1186/s12874-022-01800-9

**Published:** 2022-12-23

**Authors:** Alrik Thiem, Lusine Mkrtchyan, Zuzana Sebechlebská

**Affiliations:** Faculty of Humanities and Social Sciences, University of Lucerne, Lucerne, Switzerland

**Keywords:** Boolean algebra, Coincidence Analysis (CNA), Combinational Regularity Analysis (CORA), Configurational comparative methods (CCMs), Multi-output optimization, Qualitative Comparative Analysis (QCA), Switching circuit theory

## Abstract

**Background:**

Modern configurational comparative methods (CCMs) of causal inference, such as Qualitative Comparative Analysis (QCA) and Coincidence Analysis (CNA), have started to make inroads into medical and health research over the last decade. At the same time, these methods remain unable to process data on multi-morbidity, a situation in which at least two chronic conditions are simultaneously present. Such data require the capability to analyze complex effects. Against a background of fast-growing numbers of patients with multi-morbid diagnoses, we present a new member of the family of CCMs with which multiple conditions and their complex conjunctions can be analyzed: Combinational Regularity Analysis (CORA).

**Methods:**

The technical heart of CORA consists of algorithms that have originally been developed in electrical engineering for the analysis of multi-output switching circuits. We have adapted these algorithms for purposes of configurational data analysis. To demonstrate CORA, we provide several example applications, both with simulated and empirical data, by means of the eponymous software package CORA. Also included in CORA is the possibility to mine configurational data and to visualize results via logic diagrams.

**Results:**

For simple single-condition analyses, CORA’s solution is identical with that of QCA or CNA. However, analyses of multiple conditions with CORA differ in important respects from analyses with QCA or CNA. Most importantly, CORA is presently the only configurational method able to simultaneously explain individual conditions as well as complex conjunctions of conditions.

**Conclusions:**

Through CORA, problems of multi-morbidity in particular, and configurational analyses of complex effects in general, come into the analytical reach of CCMs. Future research aims to further broaden and enhance CORA’s capabilities for refining such analyses.

**Supplementary Information:**

The online version contains supplementary material available at 10.1186/s12874-022-01800-9.

## Background

Configurational comparative methods (CCMs), the currently most sophisticated of which are Qualitative Comparative Analysis (QCA; [1–3]) and Coincidence Analysis (CNA; [4, 5]), have begun to evolve during the 1980s in sociology and political science for inferring about cause-effect relations from configurational data. The structure of these relations is defined by the so-called INUS Theory [6–8], which enjoys a long intellectual pedigree in the philosophy of causation [9, 10] (an “INUS condition” is an insufficient but non-redundant part of an unnecessary but sufficient condition). For identifying INUS structures, which are represented in the formal language of propositional logic, CCMs employ optimization algorithms that operate on Boolean-algebraic functions [11–14].

Since the late 2010s, CCMs have also started to make inroads into medical and health research. For example, QCA has been used in pediatrics to analyze the effect of social networks on lice infestation among Mexican children [15], in ophthalmology to investigate the association between dietary patterns and macular degeneration [16], and in obstetrics for studying the effect of family policies and public health initiatives on breastfeeding initiation [17]. According to a recent review, QCA has been used so far in at least 26 studies to analyze public health interventions [18]. CNA has been employed, for instance, to examine the strategies that Veteran Affairs sites use for implementing new hepatitis C treatments [19]. In addition, the method has recently been introduced in implementation science [20].

Another development that set in about 25 years ago in medical and health research concerns the increasing relevance of the concept of multi-morbidity, which is generally defined as the co-occurrence of at least two chronic or acute conditions in patients [21]. Many numerical indicators attest to the growing need of devoting attention to problems of multi-morbidity. According to one study, 65 percent of aged Medicaid beneficiaries in the United States had suffered from multiple chronic conditions towards the end of the 1990s already [22]. By the early 2010s, adults with multiple chronic conditions became the major users of health care services at all adult ages and accounted for more than two-thirds of health care spending [23], a trend that has been projected to persist [24]. Reflective of these developments, journals such as Health Psychology have also published special issues on topics around multi-morbidity [25]. In short, multi-morbidity represents a problem of growing concern in medical and health research. Consequentially, the need for analytical methods suitable for the scientific study of data from contexts of multi-morbidity continues to increase.

Existing CCMs still face limitations in this connection. Neither QCA nor CNA offer the possibility to analyze complex effects, and neither method is thus able to correctly analyze multiple conditions and their possible conjunctions simultaneously. On the one hand, this should come as no surprise because the INUS Theory has so far focused on the complexity of causes, but not the complexity of effects. On the other hand, some prominent clinical psychologists had already pointed out the close connection between configurational thinking in terms of INUS causation and the intensifying problem of multi-morbidity about twenty years ago [26]. An initiation of efforts to incorporate the notion of complex effects into current configurational methodology and the INUS Theory thus appears a long overdue and worthwhile undertaking.

Against the background of fast-growing numbers of patients with multi-morbid diagnoses and the acknowledged yet untapped analytical potential of CCMs in this regard, the present article introduces a new method with which data from contexts of multi-morbidity can be modelled configurationally. We call this new method Combinational Regularity Analysis (CORA). In addition to its technical innovations that allow the simultaneous analysis of multiple conditions and their conjunctions under a coherent inferential framework, CORA introduces the possibility to mine configurational data and to visualize results by means of logic diagrams. The eponymous software package CORA [27] brings all these procedures together and makes them available for application by the scientific community.

## Methods

In the first subsection, we briefly revisit the state of the art in configurational data analysis with QCA and CNA, with an emphasis on the type of causal structures these methods are able to identify. Furthermore, we recapitulate basic inference requirements for CCMs. In this connection, we also discuss problems that currently arise when working with multiple effects. In the second subsection, we introduce the notion of the multi-output switching circuit and define all relevant concepts. For bridging the gap to configurational causal inference under the INUS Theory, we also translate these concepts into CCMs’ language of propositional logic. This can be done with ease as propositional logic and switching circuit theory (and also set theory) are equivalent branches of the same underlying Boolean algebra (see [28] for a concise overview). In the third subsection, we present logic diagrams as a useful device for visualizing complex configurational cause-effect relations. In the fourth section, we briefly explain the data-mining feature of CORA. In the fifth and final section, the software package CORA is introduced.

### Configurational State of the Art

US sociologists Kriss Drass and Charles Ragin have developed QCA in the mid-1980s [29, 30]. By importing the so-called Quine-McCluskey algorithm (QMC) from electrical engineering into the social sciences, their major—yet initially unintended—accomplishment was to find a functional procedure that could operationalize the central ideas of the INUS Theory. As it turned out, the second phase of the two-phase protocol of QMC also solved the so-called "Manchester-Factory-Hooters Problem", which had stood in the way of a broader acceptance of the INUS Theory until then [14]. In this way, QCA has not only provided a new lease of life to the INUS Theory, which by that time had been marginalized in the literature on the philosophy of causation [31], but it has also reverberated more generally throughout the area of social research methodology [32, 33].

Regardless of its early achievements in the social sciences, QCA has always remained restricted to the simple analysis of exactly one effect, usually called "outcome" in configurational parlance [34]. Although some tentative attempts at loosening this restriction have been made [35], the possibility that data may contain evidence for the existence of more than one outcome, not to mention the question of how such data could be adequately analyzed, has never been put on QCA’s methodological agenda. This stagnation in the development of the method’s analytical capabilities cannot be due to the fact that hardly any set of data features more than one possible outcome. In fact, many QCA studies have analyzed several distinct yet clearly co-occurring outcomes as part of the same set of data (e.g., [36–40]).

CNA has attempted to relax the restriction to single outcomes from the beginning by adding an analytical step to those performed in QCA: for each outcome that the method has identified a possible solution for, called atomic solution formula (ASF), CNA seeks to conjunctively combine these formulae into a so-called complex solution formula (CSF). CSFs can take on the form of a causal-chain structure or a common-cause structure. In the former, at least one effect features as a cause to at least one other effect. In the latter, at least one cause features as a cause to at least two effects. Although its developers have emphasized that CNA is custom-built for analyzing causal structures with multiple outcomes [5], the method still operates within the same limits as QCA with regard to the complexity of effects. The option to analyze multiple outcomes clearly represents an advantage over QCA, but CNA continues to treat outcomes in complete isolation from each other. It does not allow for the possibility that effects—not only causes—may interact in complex ways.

Besides clarifying the general structure of relations both QCA and CNA can identify—complex causes, simple effects—it is important to revisit the basic requirements for configurational causal inference. Under the INUS Theory, any potential cause must be a Boolean difference-maker to its effect: a cause must, at the very least and *ceteris paribus*, be a consistent concomitant of its effect while the absence of that cause must be a consistent concomitant of the absence of its effect [6]. If a candidate for a cause occurs, *ceteris paribus*, in conjunction with the analyzed effect as well as the absence of that effect, it can never be a difference-maker to that effect. If it is no difference-maker, it is redundant. Any causal explanation of an effect must therefore be functionally minimal, in the sense that all redundancies must have been eliminated beforehand. More specifically, every QCA solution and every ASF in CNA must be a Boolean expression representing a minimally necessary disjunction of minimally sufficient conjunctions in order to be causally interpretable [41, 42]. Such a disjunction is then usually called a model. The process of Boolean optimization, which can be carried out in very different algorithmic ways [13], seeks to ensure the generation of such models.

After having summarized the structure of causal relations QCA and CNA can identify and the general foundations of configurational causal inference, we next need to sensitize readers to a relatively unknown problem in multi-outcome analyses with CNA: the so-called "causal-chain problem" [43]. Although it has received virtually no shrift so far in the literature, a closer look turns out to be a perfect didactic stage setter for CORA. The gist of the problem is that no causal chain is ever strictly identifiable because every chain-type CSF can be transformed, by simple syntactical substitution, into an equivalent common-cause-type CSF that does not feature chain-type elements any longer. Put differently, it is impossible for CNA to ever unambiguously identify a causal chain. While disadvantageous, the non-identifiability of causal chains per se does not seem to create any deeper problems. Yet, what seems to be a minor inferential downside at first turns out, at closer inspection, to create major first-order disturbances for the requirement of functional minimality.

As an example of this problem, consider the causal chain identified by CNA in [44] in Expression 1 (for simplicity but without loss of generality, all complications which are of no relevance for the ensuing argument have been dropped):

1 $$\begin{aligned} \left( l^{\prime }\cdot t^{\prime } + s \Leftrightarrow x\right) \cdot \left( x + t \Leftrightarrow m\right) , \end{aligned}$$

where the italicized letters *l*, *t*, *s*, *x* and *m* (and all italicized letters in the remainder of this article) stand for propositional variables taking on specific values (the substantive meaning of *l*, *t*, *s*, *x* and *m* is irrelevant), “$\,'\,$” symbolizes the logical concept “not”, formally called negation, “$\,\cdot \,$” stands for the logical concept “and”, formally called conjunction, “$+$” for the logical concept “or”, formally called disjunction, and “$\Leftrightarrow$” for the logical concept “if, and only if,”, formally called equivalence. A literal is an occurrence of a propositional variable, either negated or not negated. As usual, in the remainder, we will drop the and-operator, “$\,\cdot \,$”, if no risk of confusion exists. In both QCA and CNA, a wide variety of other syntactical symbols and conventions is often used. In the remainder of this article, we stick to the above nomenclature in relation with the use of CCMs because of its compactness.

As *x* features not only as an effect, but also as a cause of *m* in Expression 1, we can transform, by direct substitution of *x* in the ASF of *m*, the causal-chain CSF into the common-cause CSF shown in Expression 2:

2 $$\begin{aligned} \left( l^{\prime }t^{\prime } + s\Leftrightarrow x\right) \left( l^{\prime }t^{\prime } + s + t\Leftrightarrow m\right) . \end{aligned}$$

**Fig. 1:**
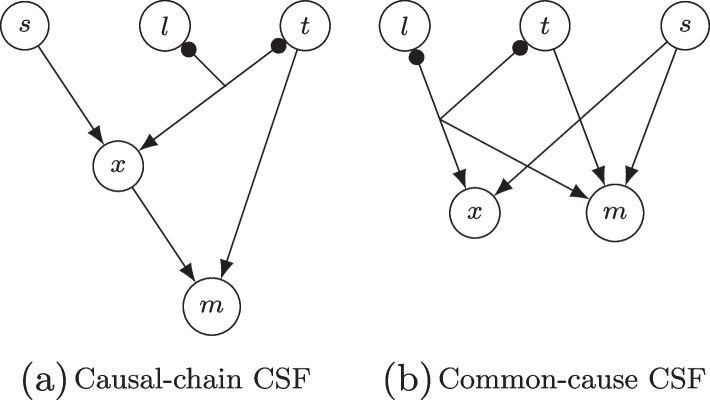
A causal-chain CSF [Expression 1] and its equivalent common-cause CSF [Expression 2]

Both CSFs are also presented graphically in Fig. 1, the causal-chain CSF in panel (a), the equivalent common-cause CSF in panel (b). Black dots at the outgoing end of a line indicate negation, joining lines conjunction, and arrows (minimal) sufficiency. This substitution process, however, brings to light an obvious redundancy in the ASF of *m* in Expression 2, in consequence of which the CSF loses its causal interpretability. More precisely, literal $t^{\prime }$ is redundant, as proven in Expressions 3a to 3c:

3a $$\begin{aligned} l^{\prime }t^{\prime } + t{} & {} = \left( t + l^{\prime }\right) \left( t + t^{\prime }\right) \quad \text {by commutativity and distribution,} \end{aligned}$$

3b $$\begin{aligned}{} & {} = \left( t + l^{\prime }\right) \left( 1\right) \quad \quad \quad \quad \quad \quad \text {by complementarity,} \end{aligned}$$

3c $$\begin{aligned}{} & {} = t + l^{\prime } \quad \quad \quad \quad \quad \quad \quad \quad \quad \quad \text {by identity.} \end{aligned}$$

Instead of a formal demonstration of redundancy, one could also approach the problem from the perspective of configurational causal inference under the INUS Theory: in order to assign $t^{\prime }$ the status of a Boolean difference-maker in conjunction with $l'$, *m* must not occur in conjunction with $l't$. However, if *t* alone is already sufficient for *m*, by extension, so must be $l't$. Put differently, if *t* alone is inferred to be a cause of *m*, it is impossible to ever infer at the same time that $t^{\prime }$ is a cause of *m* in conjunction with $l'$.

To ensure redundancy-freeness, CNA therefore eliminates $t^{\prime }$ from the ASF of *m* in the common-cause CSF in Expression 2, but does not further manipulate the corresponding chain CSF. Thus, the question arises whether such unwanted redundancies are an exclusive problem of common-cause CSFs. After all, it seems as if the problematic redundancy has been induced by the very process of substitution. That, however, is a false impression. In fact, the redundancy has already been present, albeit less obviously so, in the chain CSF. To prove this, there are several routes. One is to demonstrate that the original chain CSF in Expression 1 and the redundancy-affected common-cause CSF in Expression 2 are, in fact, strictly identical. We provide such a proof of identity in Additional file 1: Appendix.

Over the following subsections, we argue that the indiscriminate elimination of all redundancies, as currently demanded in CNA, does not provide an adequate solution for restoring causal interpretability once configurational analyses move beyond the study of single effects. Instead, the current approach to configurational data analysis must be generalized to consistently absorb them. What we show is that such a generalization has already been proposed in concept more than 50 years ago in a field that has not had any place in CNA’s development, and whose contribution has never received due recognition in QCA despite QCA’s heavy reliance on QMC. The field we allude to is that of electrical engineering.

In the remainder of this article, we will demonstrate that the relevance of electrical engineering extends far beyond the use of QMC in QCA. In fact, we have chosen the name Combinational Regularity Analysis (CORA) for our new method because that subfield of electrical engineering from which we import most of our procedures is called "combinational circuit design". “Regularity”, on the other hand, indicates CORA’s firm anchoring in the group of regularity accounts of causation, to which also the INUS Theory belongs [9].

### Multi-Output Switching Circuits

Electrical engineering is centrally concerned with building switching circuits for operating digital devices. At the most basic level, these circuits consist of switches working in parallel, switches working in series, and inverters that open a closed switch and close an open switch, respectively. Parallel switches are implemented through so-called OR-gates: it is sufficient to activate at least one of the switches to close the circuit. Serial switches, in contrast, are implemented through AND-gates: all switches need to be activated to close the circuit. For instance, every domestic appliance with an on-off-switch and a safety switch to protect children from accidents contains, in one form or another, a serial circuit component.

The mathematical framework for analyzing the conversion of a given set of input signals to a desired set of output signals in order to make a circuit perform according to a prespecified behavior is provided by the algebra of switching circuits, a branch of the same Boolean algebra of which also propositional logic and set theory are varieties [45, 46]. As propositional logic and switching circuit theory (and set theory) are so intimately linked, it is straightforward to translate concepts from one language to the other(s): OR-gates correspond to propositional disjunctions (and to set-theoretic unions), AND-gates to propositional conjunctions (and to set-theoretic intersections), and inversions to propositional negations (and to set-theoretic complements).

In devising more complex electrical devices, it is frequently necessary to simultaneously specify several switching functions that share the same inputs (because there is no risk of confusion, we will drop the addition “switching” in “switching function” from now on). Such a set of functions is called a system of functions. As more than one possible circuit layout usually fulfills the desired specification, the optimization of multi-output circuits is an important stage in the design process of a switching circuit [47, 48]. Encoders and decoders, for example, are generic applications.

One of the most crucial questions electrical engineers have to address in the process of designing a circuit concerns the optimization of its hardware infrastructure. More specifically, given two different circuits that produce the same outputs when provided with the same set of inputs, the circuit demanding less costly infrastructure is preferred. More formally and generally, this problem can be phrased as follows:

- **Central Problem of Multi-Output Optimization**: Given a system of functions $\textbf{F} = \{f_{1}\left( \textbf{x}\right) , f_{2}\left( \textbf{x}\right) , \ldots , f_{m}\left( \textbf{x}\right) \}$ and an objective function $\mathcal {O}$ defined on the set of $\textbf{F}$-equivalent systems $\textbf{S}_{\textbf{F}}$, what is the set $\mathrm {\textbf{S}}^{*}_{\textbf{F}} \in \textbf{S}_{\textbf{F}}$ for which $\mathcal {O}$ reaches an optimum?

Potentially, there are many ways in which $\mathcal {O}$ could be defined. It can relate to the number of gates, gate contacts, or a multi-dimensional requirement of the form $aP + bQ + cR$, where *P*, *Q*, and *R* represent the number of gates of a certain type and *a*, *b* and *c* are weighting coefficients on unit price, reliability or other economical or technical criteria [49].

A very common specification of $\mathcal {O}$ is called sum irredundancy, which, at least up to the late 1950s, also provided the objective function for QMC in optimizing switching circuits with single outputs. With sum irredundancy set as the objective function, the purpose of the optimization algorithm, whether QMC or else, is to find all possibilities for a circuit infrastructure that does not contain any unnecessary AND-gates [46], that is, AND-gates that are redundant in ensuring that the output of a circuit given a certain combination of inputs corresponds to the desired specification. A possible circuit layout that results from this process is correspondingly called an "irredundant sum"; “sum” because AND-gates—the first level of two-level circuits—can more generally be called Boolean products, while OR-gates—the second level—can more generally be called Boolean sums. An AND-gate that could, but not necessarily is, a component of an irredundant sum is called a prime implicant (PI).

**Fig. 2:**
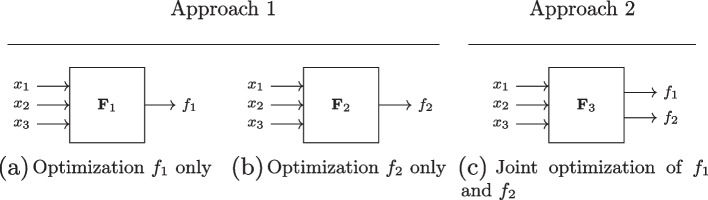
Two different approaches to optimizing a system of two functions

In contrast to single-output optimization problems, situations involving two or more outputs require additional considerations. Figure 2 shows two possible approaches to the optimization of a system of two functions: under Approach 1, the two functions $f_{1}$ and $f_{2}$ of inputs $x_{1}$, $x_{2}$ and $x_{3}$ can be optimized separately as two quasi-independent systems, $\textbf{F}_{1}$ and $\textbf{F}_{2}$, shown in panels (a) and (b), respectively. Alternatively, they can be optimized jointly as a 2-output system, $\textbf{F}_{3}$, as shown under Approach 2 in panel (c).

It may be suspected that the two approaches produce the same result, simply through different routes. However, this conjecture does not hold. The reason is that Approach 1 and Approach 2 may not generate the same set of PIs. Most importantly, under Approach 2, the complexity of a circuit’s infrastructure may regularly be reduced by explicitly searching for PIs that are shared between functions. These PIs may not be PIs in the separate optimization of each function. Moreover, PIs that do not become parts of any irredundant sum under Approach 1, called "useless" PIs, may become useful, that is, part of at least one irredundant sum, under Approach 2.

Consider the example of a system of functions $f_{1}(x,y,z) = \sum (1,3,7)$ and $f_{2}(x,y,z) = \sum (3,6,7)$ (as usual, functions are most compactly represented with decimal numbers; for instance, 1 is the decimal equivalent of $x'y'z$, 3 of $x'yz$ because in binary-number notation, 1 is expressed as 001, 3 as 011). Thus, at $x'y'z$, $x'yz$ and *xyz* it is the case that $f_{1} = 1$, and $f_{1} = 0$ otherwise; at $x'yz$, $xyz'$ and *xyz* it is the case that $f_{2} = 1$, and $f_{2} = 0$ otherwise. Any optimization algorithm with sum irredundancy set as its objective function reveals the two irredundant sums $f_{1} = x'z + yz$ and $f_{2} = xy + yz$, respectively, under Approach 1. If the corresponding circuits were built back into one system, four AND-gates and two OR-gates would thus be required. However, it is obvious in this case that $f_{1}$ and $f_{2}$ share *yz* as a PI. A circuit in which one of the corresponding AND-gates could be dispensed with would thus represent a strictly preferable alternative.

A similar yet far less obvious example involves the 2-output system of functions $f_{1}(x,y,z) = \sum (1,3,7)$ and $f_{2}(x,y,z) = \sum (2,6,7)$. In this case, the irredundant sums resulting under Approach 1 are $f_{1} = x'z + yz$ and $f_{2} = xy + yz'$, respectively. If both circuits were built, again, four AND-gates and two OR-gates would be required. More difficult to see is that the alternative single-circuit system $f_{1} = x'z + xyz$ and $f_{2} = xyz + yz'$ requires only three AND-gates because one of these gates could use *x*, *y* and *z* as joint inputs to $f_{1}$ and $f_{2}$. In contrast to the previous example, however, *xyz* is no PI of either function optimized independently because it contains redundant elements. For example, with regard to $f_{1}$, Expressions 4a to 4c provide one way of proving *x* to be redundant in *xyz*:

4a $$\begin{aligned} x'z + xyz{} & {} = x'z + xyz + yzz \quad \quad \text {by consensus,} \end{aligned}$$

4b $$\begin{aligned}{} & {} = x'z + xyz + yz \quad \quad \text {by idempotency,} \end{aligned}$$

4c $$\begin{aligned}{} & {} = x'z + yz \quad \quad \quad \quad \quad \quad \text {by absorption.} \end{aligned}$$

Respecting $f_{2}$, Expressions 5a to 5c provide one way of doing the same with regard to *z* in *xyz*:

5a $$\begin{aligned} xyz + yz'{} & {} = xyz + yz' + xyy \quad \quad \text {by consensus,} \end{aligned}$$

5b $$\begin{aligned}{} & {} = xyz + yz' + xy \quad \quad \text {by idempotency,} \end{aligned}$$

5c $$\begin{aligned}{} & {} = xy + yz' \quad \quad \quad \quad \quad \quad \text {by absorption.} \end{aligned}$$

At this stage, obvious similarities between the occurrence of redundancies in configurational data analyses of multiple outcomes with existing CCMs and the separate optimization of one system’s functions in electrical engineering already start to become noticeable. Modern CCMs search for minimally necessary disjunctions of minimally sufficient conjunctions in order to generate causally interpretable models. In switching circuit theory, PIs are what minimally sufficient conjunctions are in configurational data analysis, minimally necessary disjunctions of minimally sufficient conjunctions are what irredundant sums are for electrical engineers. As propositional logic and switching circuit theory are merely two branches of the same underlying Boolean algebra, these concepts are completely equivalent.

In electrical engineering applications, where the primary objective of functional optimization is a reduction in circuit build costs, the inclusion of redundancies results in unnecessarily high build costs because a redundant input to an AND-gate or an OR-gate does not make a difference to the required operation of the circuit. In configurational data analysis with QCA and CNA, redundancies render models returned by these methods causally uninterpretable because a redundant element can never be a Boolean difference-maker [recall the causal-chain problem above and the redundancy of literal $t^{\prime }$ in Expression 2].

Motivated by the possibility to reduce build costs through complete redundancy elimination, electrical engineers have already noticed about 60 years ago that it is inadequate to optimize each function separately when addressing problems that involve multiple outputs [46, 50–52]. In order to realize cost savings, all possible products of functions must be considered in addition to and simultaneously with each individual function. In consequence, the concept of the “prime implicant” has been generalized from the simple single-output to the multi-output framework. A PI resulting under such a framework is called a "multi-output prime implicant" (MOPI).

#### Definition 1

A multi-output prime implicant (MOPI) of a system of functions $\textbf{F} = \{f_{1}\left( \textbf{x}\right) , f_{2}\left( \textbf{x}\right) , \ldots , f_{m}\left( \textbf{x}\right) \}$ of a set of inputs $\textbf{x} = \{x_{1}, x_{2}, \ldots , x_{k}\}$ is a product of literals $x^{\{\cdot \}}_{1;i}x^{\{\cdot \}}_{2;i}\cdots x^{\{\cdot \}}_{h;i}$ with $h \le k$ and $1 \le i_{j} \le k$, which is either a PI of some $f_{j} \in \textbf{F}$ with $j = 1,2,\ldots ,m$ or a PI of one of the product functions $f_{1}\left( \textbf{x}\right) f_{2}\left( \textbf{x}\right) \cdots f_{m}\left( \textbf{x}\right)$.

**Fig. 3:**
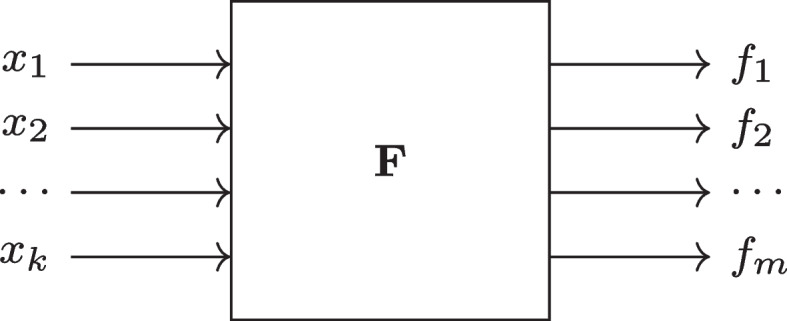
General *k*-input *m*-output system

On the basis of Definition 1, we can now also generalize Approach 2 introduced above in Fig. 2. Diagrammatically sketched in Fig. 3, any system of functions $\textbf{F}$ can potentially have *k* inputs and *m* outputs. For $m > 1$, PIs become MOPIs.

If, for multi-output optimization problems, redundancies must be made room for, the crucial question then is how to ensure that the switching circuit is most efficient according to the objective function $\mathcal {O}$, that the result of Boolean optimization in configurational data analysis remains causally interpretable, respectively. Above, we have seen that the requirement of absolute redundancy elimination can create problems because the generation of minimally sufficient conjunctions with respect to one outcome may no longer remain minimally sufficient beyond that single outcome. Electrical engineers have also solved this problem by elevating the concept of irredundancy from the level of simple functions to the level of systems of functions [51].

#### Definition 2

An $\textbf{F}$-equivalent system of functions $S \in \textbf{S}_{\textbf{F}}$ is called an irredundant system $S^{*} \in \textbf{S}^{*}_{\textbf{F}}$ if it is impossible to cancel any literal in the writing of its MOPIs and any MOPI in the writing of its functions $f_{j}$ and still be able to ensure $\textbf{F}$-equivalence.

Definition 2 leaves it open whether a process of Boolean optimization results in only one irredundant system, two systems, a dozen or hundreds of systems. It is well possible—and usually the rule rather than the exception—that multiple irredundant systems represent potential candidates for a circuit’s infrastructure. Without any further criteria, none of these systems is preferable to another because they all comply with the objective function of sum irredundancy.

In configurational data analysis with QCA or CNA, the existence of multiple models that fit the data equally well has been referred to as "model ambiguity" [14, 53, 54]. Under the multi-output approach of CORA, we will speak of "systems ambiguity" instead because each system comprises as many models as there are outputs, but these models are not alternatives to each other, whereas different systems are. To put this observation on a formal footing, we further introduce the concept of the solution to CORA in Definition 3.

#### Definition 3

A solution $\mathcal {S}$ is the set of all irredundant systems $\textbf{S}^{*}_{\textbf{F}}$.

At this stage, we have all necessary theoretical concepts in place. In the following subsection, we introduce a core feature of CORA that has also been imported from electrical engineering: logic diagrams.

### Logic Diagrams

Irrespective of how carefully a research design has been constructed and of how sophisticated the employed method is, if results cannot be communicated effectively, the impact of a study may be reduced considerably. Thus, graphics and visualization have played an increasing role in conveying the results of scientific work. So far, neither QCA nor CNA have offered consistent means of visualization. Depending on software, academic discipline, and personal preferences, researchers have used Venn diagrams, bivariate scatter plots, Tosmana maps and numerous other means for communicating their findings [55].

In contrast to QCA and CNA, CORA offers an established and standardized means for communicating its results graphically: logic diagrams. Initially, these diagrams have been developed by electrical engineers to visualize the architecture of switching circuits, but according to Judea Pearl, these diagrams also capture “in my opinion, the very essence of causation” [56]. Despite their apparent usefulness, however, only very few scientific disciplines in which causal inference plays a central role have so far adopted logic diagrams [57, 58].

**Fig. 4:**
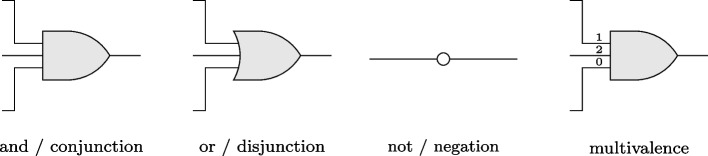
Basic logic symbols used in CORA

A common standard for the production of logic diagrams is provided by MIL-STD-806B, a document that establishes uniform engineering and technical requirements for military or commercial processes, procedures, practices, and methods [59]. For two-level circuits, three core elements of this standard suffice: one for the and-operator / conjunction, one for the or-operator / disjunction, and one for the not-operator / negation. If multivalent inputs and outputs, that is, factors having more than two levels, should be allowed as well, level indicators must be added. These four elements, which together make up the graphical repertoire of logic diagrams in CORA, are shown in Fig. 4.

**Fig. 5:**
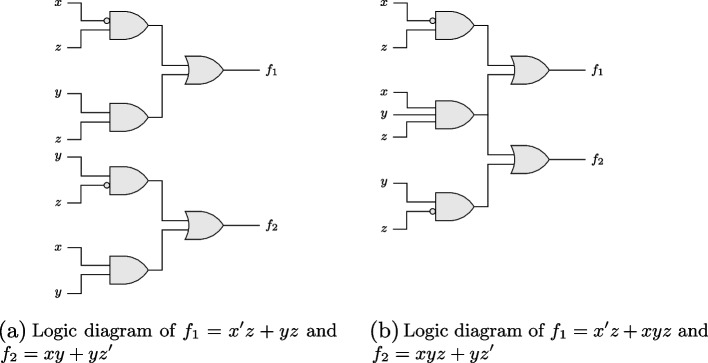
Two examples of a logic diagram

For example, consider the case of the 2-output system of functions $f_{1}(x,y,z) = \sum (1,3,7)$ and $f_{2}(x,y,z) = \sum (2,6,7)$ discussed above in relation to Expressions 4a to 4c and 5a to 5c. Under an approach of separate optimization, $f_{1} = x'z + yz$ and $f_{2} = xy + yz'$ result as the two corresponding irredundant sums. Their respective circuits are visualized in the logic diagrams in panel (a) of Fig. 5. In contrast, the alternative single-circuit system of functions $f_{1} = x'z + xyz$ and $f_{2} = xyz + yz'$ that results under joint optimization is visualized in panel (b).

### Data Mining

Besides the possibility to analyze configurational multi-output problems and to visualize results by means of logic diagrams, a third advantage of CORA over QCA and CNA is the option to mine data. The basic idea behind this approach is that any system that is found with a given number of inputs, must, *ceteris paribus*, also always be found in an analysis with only those inputs present in the system. For example, if a solution includes a system that consists only of inputs $x_{1}, x_{3}, x_{5}$, in whatever constellation, following an optimization process involving the input set $\textbf{x}_{a} = \{x_{1}, x_{2}, x_{3}, x_{4}, x_{5}\}$, then this system should also be found following an optimization process involving the reduced input sets $\textbf{x}_{b} = \{x_{1}, x_{2}, x_{3}, x_{5}\}$ or $\textbf{x}_{c} = \{x_{1}, x_{3}, x_{4}, x_{5}\}$ or $\textbf{x}_{d} = \{x_{1}, x_{3}, x_{5}\}$.

Although the basic idea behind this approach to input selection has first been tested in the context of QCA [60, 61], CORA is the first CCM to offer an in-built and systematic possibility to apply a tuple selection procedure. If, for example, a researcher has four potential inputs $\textbf{x} = \{x_{1}, x_{2}, x_{3}, x_{4}\}$ available for inclusion, CORA can be asked to test whether the inclusion of $\textbf{x} = \{x_{1}\}$ alone or $\textbf{x} = \{x_{2}\}$ alone or $\textbf{x} = \{x_{3}\}$ alone or $\textbf{x} = \{x_{4}\}$ alone suffices to generate a solution that meets the researcher’s criteria. If unsuccessful, CORA proceeds to tuples of two, i.e. $\textbf{x} = \{x_{1},x_{2}\}$, $\textbf{x} = \{x_{1},x_{3}\}$, and so on. From this perspective, CORA’s data-mining approach represents a type of Occam’s Razor, which says that explanations that involve fewer variables are, *ceteris paribus*, to be preferred over explanations that are more complex. Note that this is not tantamount to setting the objective function in Boolean optimization to what is called "sum minimality". A minimal sum is that irredundant sum which has the smallest number of PIs, but not necessarily the smallest number of inputs.

Not least of all, there are additional practical considerations that motivate the option of data mining. Often, researchers have more variables available than can reasonably be included in a configurational analysis. For example, in one study on the effectiveness of health promotion networks, the authors have identified no fewer than 42 potential determinants of effectiveness while having only 13 cases of health promotion networks [62].

Moreover, the more inputs researchers feed into the optimization process given a fixed number of cases, the higher their measure of fit statistics tend to become, but the higher the degree of model ambiguity also becomes. The relationship between the number of inputs and the number of models in a QCA or CNA solution has not yet been systematically studied, but existing data experiments suggests that beyond four inputs, model ambiguity starts to become the rule rather than the exception and tends to increase in severity with every additional input [53]. For instance, a recent meta analysis of 215 peer-reviewed QCA articles from across 109 management, political science and sociology journals found that one in three QCA studies was affected by (unreported) model ambiguity, one in ten severely so [14]. Absent other means of ranking multiple and equally well-fitting systems, the option of data mining provides researchers with a practical way to achieve a reduction in systems ambiguity.

### Software

**Fig. 6:**
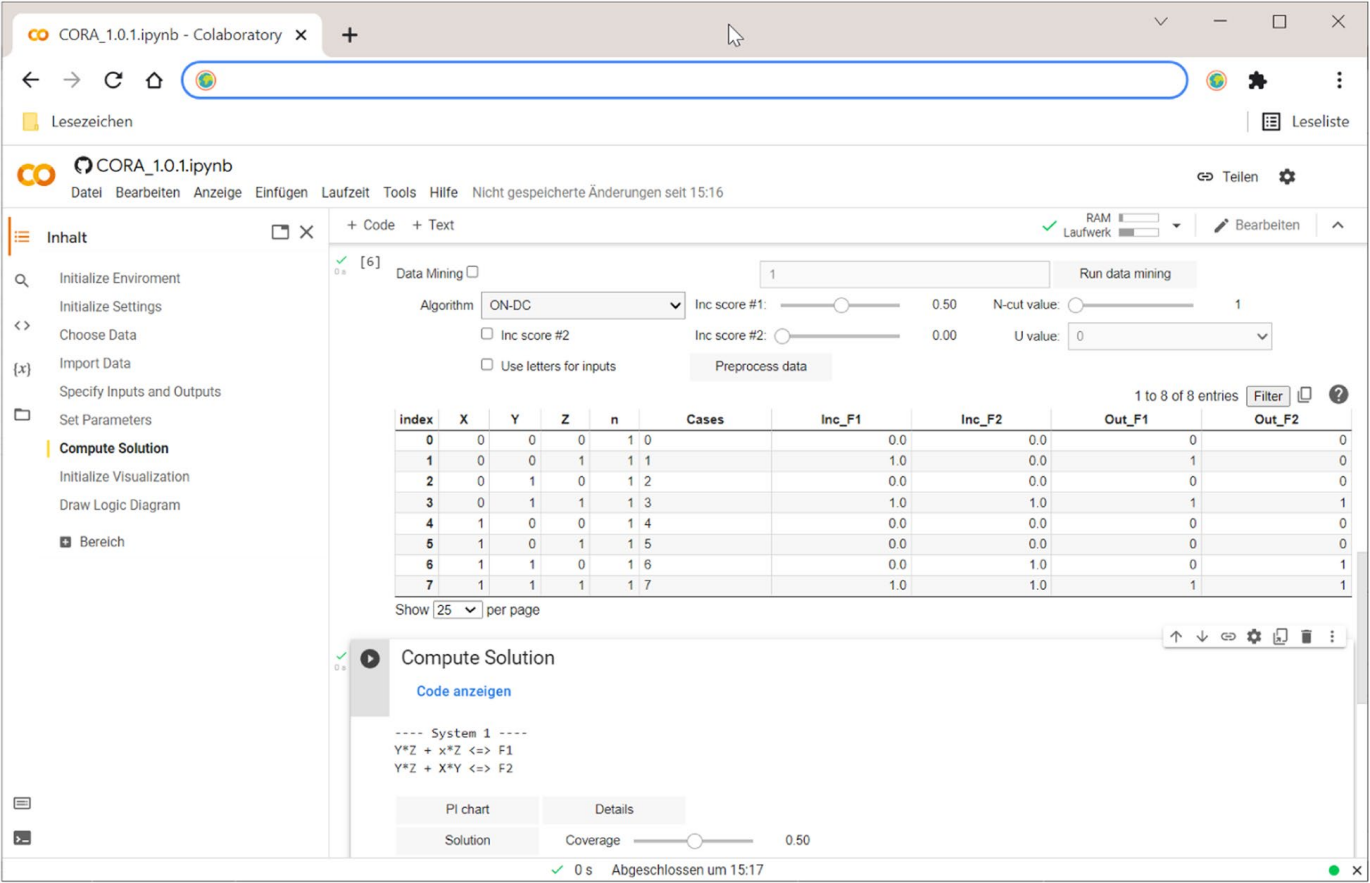
Interface of Software CORA

Methods and algorithms can be theoretically developed and also methodologically evaluated, but without appropriate software, they have no value to applied researchers. All procedures described above, plus additional ones, have thus been made available to the scientific community in the open-source Python/C++ package CORA [27], a screenshot of whose interface is shown in Fig. 6.

The workflow in CORA is pre-determined to guide users through the analysis. It comprises nine steps, the last two of which are optional: (1) the initialization of the framework and (2) default settings, (3) the choice and (4) import of data, (5) the specification of the inputs and outputs, (6) the setting of search parameters and thresholds for data fit statistics, (7) the computation of the solution, (8) the initialization of CORA’s visualization module and finally (9) the drawing and export of logic diagrams. In CORA, logic diagrams are integrated via a stand-alone visualization module called LOGIGRAM [63]. Accordingly, the particular form of logic diagram generated in CORA is called a “logigram”. For reasons of space, we cannot introduce CORA in detail here. This will be done in a separate software tutorial.

## Results

In this section, we provide four basic example applications of CORA. The first example uses a relatively simple set of artificial data, the second example provides a showcase analysis of data from a typical context of multi-morbidity, the third example uses a more complex set of artificial data, and the fourth example is taken from a multi-outcome QCA study that analyses the impact of structural factors on the injury rate in 12 European countries [64].

### Example 1: Artificial data; simple structure

In this first example, we use artificial data on a simple multi-output problem, to which numerous basic applications could potentially fit. The main objective is not to generate any substantive insights, but to demonstrate the generic workings of CORA and to describe how the method’s output has to be interpreted.

To the three inputs *x*, *y*, and *z*, consider the following system of two functions $f_{1}$ and $f_{2}$ given in Expressions 6a to 6b:

6a $$\begin{aligned} f_{l}\left( x, y, z\right){} & {} = \sum {m\left( 1,3,7\right) } \end{aligned}$$

6b $$\begin{aligned} f_{2}\left( x, y, z\right){} & {} = \sum {m\left( 2,6,7\right) } \end{aligned}$$

Under a certain combination of inputs, namely *xyz* (term 7), both outputs are present. Under all other combinations, either only one output is present or none. From an applied perspective, data of this structure may signal that the simultaneous presence of some combination of the risk factors *x*, *y* and *z* is responsible for the simultaneous presence of medical conditions $f_1$ and $f_2$. If these data were analyzed with QCA or CNA, each would find output $f_1$ to be caused by $x'z$ or *yz* and output $f_{2}$ by *xy* or $yz'$. They would not find any commonalities between these two outputs.

With CORA, an analysis of complex effects is straightforward. For the given data, CORA’s solution consists of two irredundant systems, as shown in Expression 7: $S^{*}_{1}$ reveals the complex cause *xyz* of the complex effect $f_{1}f_{2}$. Once this complex effect is explained, individual causes of $f_{1}$ alone, $f_{2}$ alone, respectively, remain. Under $S^{*}_{1}$, CORA identifies $x'z$ for $f_{1}$ alone, $yz'$ for $f_{2}$ alone, respectively. Alternatively, there may not be any common cause, but each effect is brought about by distinct causes. $S^{*}_{2}$ reveals this alternative possibility, which is identical with the result QCA and CNA generate.

7 $$\begin{aligned} \mathcal {S} = \left\{ \begin{array}{cc} S^{*}_{1} = &{} \left\{ \begin{array}{c} xyz + x'z \Leftrightarrow f_{1}\\ xyz + yz' \Leftrightarrow f_{2} \end{array}\right. \\ S^{*}_{2} = &{} \left\{ \begin{array}{c} yz + x'z \Leftrightarrow f_{1}\\ xy + yz' \Leftrightarrow f_{2} \end{array}\right. \\ \end{array}\right. \end{aligned}$$

This simple example illustrates in a direct way why CORA’s inferential capabilities extend beyond those of QCA and CNA. If there are indeed no complex effects to be explained, CORA will detect this possibility in the same way as QCA or CNA will. However, if common causes to complex effects exist, CORA will be the only method that can reveal this possibility because it operates under a system-level conception of irredundancy, whereas both QCA and CNA are restricted to an output-level conception of irredundancy.

### Example 2: Applied example on multi-morbidity

In this example, we illustrate the potential of CORA for problems related to the study of multi-morbidity. Table 1 shows data on eleven patient groups $p_1$ to $p_{11}$. The first four columns contain information on four socio-demographic and economic characteristics, namely gender, income (level), family history (of depression or diabetes) and marital status. The last two columns show data on two health conditions, namely diabetes and depression.

Although the data in Table 1 have been purposefully chosen for demonstration purposes, several studies point towards strong relationships within such data. For instance, it has been argued that “depression comorbid with other chronic diseases produced significantly greater decrements in health than from one or more chronic diseases, and that this additive effect is substantially amplified in the case of depression comorbid with diabetes” [65]. Other studies have shown striking associations between socio-economic and socio-demographic factors, and chronic diseases such as depression and diabetes [66, 67]. Last, but not least, a significant body of epidemiologic studies emphasizes that a positive family history increases the risk among first-degree relatives for diabetes [68].

**Table 1:** Socio-demographic factors of eleven patient groups for two health conditions: diabetes and depression^a^

Patient Group	Inputs				Outputs	
	*g*	*i*	*f*	*m*	*a*	*e*
$p_{1}$	1	0	1	1	1	1
$p_{2}$	1	0	1	0	1	1
$p_{3}$	0	1	0	0	0	0
$p_{4}$	0	0	1	1	1	1
$p_{5}$	1	1	1	0	1	1
$p_{6}$	1	1	1	1	1	1
$p_{7}$	1	0	0	1	0	1
$p_{8}$	0	1	1	0	1	1
$p_{9}$	0	0	0	0	1	1
$p_{10}$	0	0	0	1	0	1
$p_{11}$	0	1	0	1	0	1

^a^ *g* = gender (1: male / 0: female); *i* = income (1: high / 0: low); *f* = family history (1: yes / 0: no); *m* = marital status (1: married / 0: single); *a* = diabetes (1: yes / 0: no); *e* = depression (1: yes / 0: no)

CORA’s solution to these data is given in Expression 8. It says that a co-morbid condition of diabetes and depression has at least two (complex) causes, the first of which contains low income and the status of being married, and the second of which contains a family history of diabetes or depression. For depression without diabetes, the status of being married is by itself part of the explanation. Again, we do not seek to interpret these findings substantively. Our primary goal here is only to explain how CORA’s findings are to be read.

8 $$\begin{aligned} \mathcal {S} = \left\{ \begin{array}{l} i'm + f \Leftrightarrow a\\ i'm + f + m \Leftrightarrow e \end{array}\right. \end{aligned}$$

In this connection, it is important to add two further notes. First, there is no empirical evidence for the causal relevance of gender for diabetes or depression, or a co-morbid condition. More generally, it must be emphasized that only because some input is not contained within CORA’s solution, this does not mean that the respective input is generally causally irrelevant to the analyzed output(s). It just means that either the input is indeed irrelevant or the data do not contain sufficient information to reveal the input as causally relevant when it is truly relevant.

Second, the data in Table 1 are such that every diabetic patient has also depression, but the opposite does not hold. There are patients who have depression but are not diagnosed with diabetes. This association of the two outputs is correctly reflected only through CORA’s generalized process of multi-output optimization. If the analyst had used QCA or CNA, the two outputs would have had only one cause in common, namely a family history of depression or diabetes. However, since the set of patient groups with diabetes is a proper subset of the set of patient groups with depression, every complex cause of diabetes must also be a part of a potential causal explanation of depression. Under the restricted notion of irredundancy in QCA or CNA, input *m* could never appear alongside $i'$ in an explanation of depression because under the Boolean theorem of absorption $i'm + m = m$.

### Example 3: Artificial data; complex structure

In this third example, we increase the complexity of the data by adding one more input as well as another output. To the four inputs *a*, *b*, *c* and *d*, consider the following system of three functions $f_{1}$, $f_{2}$ and $f_{3}$ given in Expressions 9a to 9c:

9a $$\begin{aligned} f_{l}\left( a, b, c, d\right)= & {} \sum {m\left( 2,3,5, ,7,8,9,10,11,13, ,15\right) } \end{aligned}$$

9b $$\begin{aligned} f_{2}\left( a, b, c, d\right)= & {} \sum {m\left( 2,3,5,6,7,   ,10,11, ,14,15\right) }\end{aligned}$$

9c $$\begin{aligned} f_{3}\left( a, b, c, d\right)= & {} \sum {m\left(      ,6,7,8,9,     ,13,14,15\right) } \end{aligned}$$

We have aligned all common input combinations visually so that it becomes easier to see which outputs have which input combinations in common. Outputs $f_1$ and $f_2$ co-occur for input combinations 2, 3, 5, 7, 10, 11 and 15; outputs $f_2$ and $f_3$ co-occur for input combinations 6, 7, 14 and 15; outputs $f_1$ and $f_3$ co-occur for input combinations 7 to 9, 13 and 15; and for input combinations 7 and 15, all three outputs co-occur. For these data, CORA’s solution consists of five irredundant systems, as shown in Expression 10:

10 $$\begin{aligned} \mathcal {S} = \left\{ \begin{array}{ll} S^{*}_{1} = &{} \left\{ \begin{array}{ll} ab'c' + a'bd + bcd + ac'd + b'c \Leftrightarrow f_1\\ a'bd + bcd + bc + b'c \Leftrightarrow f_2\\ ab'c' + bcd + ac'd + bc \Leftrightarrow f_3\\ \end{array}\right. \\ S^{*}_{2} = &{} \left\{ \begin{array}{ll} ab'c' + a'bd + ac'd + b'c + ad \Leftrightarrow f_1\\ a'bd + bc + b'c \Leftrightarrow f_2\\ ab'c' + ac'd + bc \Leftrightarrow f_3\\ \end{array}\right. \\ S^{*}_{3} = &{} \left\{ \begin{array}{ll} ab'c' + a'bd + abd + b'c \Leftrightarrow f_1\\ a'bd + bc + b'c \Leftrightarrow f_2\\ ab'c' + abd + bc \Leftrightarrow f_3\\ \end{array}\right. \\ S^{*}_{4} = &{} \left\{ \begin{array}{ll} ab'c' + a'bd + ac'd + b'c + bd \Leftrightarrow f_1\\ a'bd + bc + b'c \Leftrightarrow f_2\\ ab'c' + ac'd + bc \Leftrightarrow f_3\\ \end{array}\right. \\ S^{*}_{5} = &{} \left\{ \begin{array}{ll} ab'c' + a'bd + ac'd + cd + b'c \Leftrightarrow f_1\\ a'bd + bc + cd + b'c \Leftrightarrow f_2\\ ab'c' + ac'd + bc \Leftrightarrow f_3\\ \end{array}\right. \end{array}\right. \end{aligned}$$

With just one more input and one more output than in the previous example, we see that the complexity of the solution may increase markedly. Instead of two systems as in Example 1, we now have five alternative systems explaining the data equally well. Each of these systems reveals a distinct possibility for a complex causal relation between the four inputs and the three outputs.

$S^{*}_{1}$ is, functionally speaking, the most complex system. It reveals several common causes of several complex effects. First, it shows that outputs $f_1$ and $f_2$ have three complex causes in common, namely $a'bd$, *bcd* and $b'c$. Second, it shows that outputs $f_1$ and $f_3$ also have three complex causes in common, namely $ab'c'$, *bcd* and $a'cd$. In contrast, outputs $f_2$ and $f_3$ co-occur as a complex effect only of *bc*. Lastly, all three outputs co-occur as a complex effect of *bcd*. As no other causes remain, no effect occurs in isolation. In applied terms, $S^{*}_{1}$ thus suggests a purely co-morbid explanation of diseases $f_1$, $f_2$ and $f_3$.

**Fig. 7:**
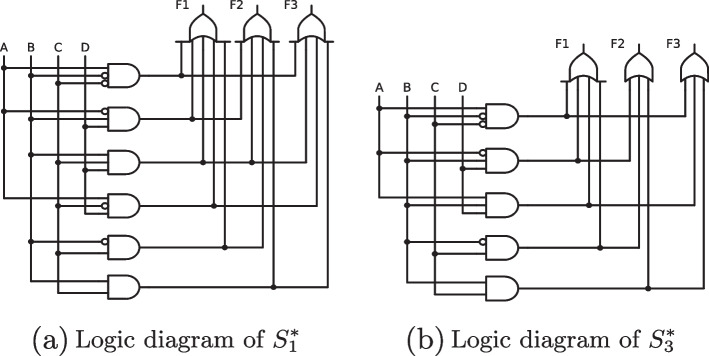
Logigrams of CORA’s solution for multi-output data in Expressions 9a to 9c

A purely co-morbid explanation is also offered by $S^{*}_{3}$. Unlike $S^{*}_{1}$, however, $S^{*}_{3}$ does not suggest any common cause of all three outputs. The extent the data can be explained through co-morbid causal relations is therefore lower than under $S^{*}_{1}$. At the same time, no effect can be explained in isolation from another effect. Only systems $S^{*}_{2}$ and $S^{*}_{4}$ include causes of isolated effects, in the former for $f_1$ (*ad*) and in the latter also for $f_1$ (*bd*). In fact, this is the only difference between these two systems. The two systems $S^{*}_{1}$ and $S^{*}_{3}$ are visualized in Fig. 7, the former in panel (a) and the latter in panel (b). Note that logigrams in CORA (currently only) allow the use of upper-case notation for inputs.

### Example 4: Empirical data on injury rates in West European countries

The aim of the study in [64] is to analyze how socioeconomic factors, such as per capita income, unemployment and alcohol consumption (which have been found to have an impact on traffic fatalities and suicides in other studies) as well as culture-oriented factors such as religion and education are related to injury mortality in 12 West European countries. Traffic injuries were chosen as the indicator for environment-related injury and suicides as that for its socially related counterpart. Table 2 shows the data for the two outputs and the five inputs of this analysis.

**Table 2:** Data on injury rates in 12 West European countries in 1990; source: [64]^a^

Country	Inputs					Outputs	
	*gnp*	*mys*	*apac*	*unem*	*roca*	*mvta*	*ssii*
Belgium	1	1	1	1	1	1	1
Denmark	1	1	1	1	0	0	1
Finland	1	1	0	0	0	0	1
France	1	1	1	1	1	1	1
Ireland	0	0	0	1	1	1	0
Italy	1	0	1	1	1	1	0
Netherlands	1	1	0	0	0	0	0
Norway	1	1	0	0	0	0	1
Portugal	0	0	1	0	1	1	0
Spain	0	0	1	1	1	1	0
Sweden	1	1	0	0	0	0	1
UK	1	1	0	0	0	0	0

^a^ *gnp* = Gross national product per capita (1: above limit / 0: below limit); *mys* = Mean years of schooling (1: above limit / 0: below limit); *apac* = Annual pure alcohol consumption (1: above limit / 0: below limit); *unem* = Unemployment rate (1: above limit / 0: below limit); *roca* = Roman Catholics percentage (1: above limit / 0: below limit); *mvta* = Age-standardized death rate for motor vehicle traffic accidents (1: above limit / 0: below limit); *ssii* = Age standardized death rate for suicides and self-inflicted injuries (1: above limit / 0: below limit)

Table 3 presents the joint truth table generated from the data in Table 2. Inclusion / consistency scores are also provided in this table. Using QCA, two separate optimization runs are necessary, one for output *mvta* and one for output *ssii*.

**Table 3:** The truth table generated from data in Table 2

*gnp*	*mys*	*apac*	*unem*	*roca*	*n*	Inc(*mvta*)	Inc(*ssii*)	f(*mvta*)	f(*ssii*)
0	0	0	1	1	1	1.0	0.0	1	0
0	0	1	0	1	1	1.0	0.0	1	0
0	0	1	1	1	1	1.0	0.0	1	0
1	0	1	1	1	1	1.0	0.0	1	0
1	1	0	0	0	5	0.0	0.6	0	1
1	1	1	1	0	1	0.0	1.0	0	1
1	1	1	1	1	2	1.0	1.0	1	1

The PI chart in Table 4 shows the list of PIs per output. It suggests that the two outputs have nothing in common because there is no shared PI. The one essential PI necessary to cover all instances of *mvta* completely is *roca*, while *mys* has the same function with respect to *ssii*.

**Table 4:** The PI chart resulting from separate optimization of the truth table in Table 3

	*mvta*					*ssii*			
	3	5	7	23	31	24	30	31	
$apac'\cdot unem$	x								$p_{1}$
$apac\cdot unem'$		x							$p_{2}$
*roca*	x	x	x	x	x				$p_{3}$
$mys'$	x	x	x	x					$p_{4}$
$gnp'$	x	x	x						$p_{5}$
$gnp\cdot apac'$						x			$p_{6}$
$gnp\cdot unem'$						x			$p_{7}$
$apac'\cdot unem'$						x			$p_{8}$
*mys*						x	x	x	$p_{9}$
$roca'$						x	x		$p_{10}$

This analysis with QCA suggests that the death rate for motor vehicle traffic accidents and the death rate for suicides and self-inflicted injuries have completely independent causes. There seems to be evidence of causal relevance only for the percentage of Roman Catholics with regard to motor vehicle accidents and only for the years of schooling with regard to suicides and self-inflicted injuries. We demonstrate these conclusions to be unsatisfactory.

**Table 5:** The PI chart resulting from multi-output optimization of the truth table in Table 3

	*mvta*					*ssii*			
	3	5	7	23	31	24	30	31	
$apac'\cdot unem$	x								$p_1$
$apac\cdot unem'$		x							$p_2$
*roca*	x	x	x	x	x				$p_3$
$mys'$	x	x	x	x					$p_4$
$gnp'$	x	x	x						$p_5$
$mys\cdot roca$					x			x	$p_6$
$gnp\cdot apac'$						x			$p_7$
$gnp\cdot unem'$						x			$p_8$
$apac'\cdot unem'$						x			$p_9$
*mys*						x	x	x	$p_{10}$
$roca'$						x	x		$p_{11}$

Table 5 shows the MOPI chart for the truth table in Table 3. MOPI charts can be generated via different algorithmic routes in CORA. Currently, users have two options, an on-dc and an on-off algorithm [69–72]. On-off algorithms enjoy significant computational advantages when the size of the dc-set is large relative to the size of the off-set, whereas on-dc algorithms enjoy significant computational advantages when the size of the off-set is large relative to the size of the dc-set, given a fixed size of the on-set. The possibility to choose between distinct yet equivalent algorithms also demonstrates that, in contrast to QCA, where researchers regularly worry about the use of logical remainders, solutions types and contradictory simplifying assumptions [73, 74], CORA is completely unaffected by such problems. Irrespective of the algorithmic choice, the objective function for deriving the complete set of irredundant systems that faithfully reflect the empirical evidence is hardwired into CORA.

Internally, CORA then applies an enhanced version of Petrick’s method to the PI chart for identifying all irredundant systems [75]. In the present replication, this process results in four such systems, which are shown in Table 6. In Expression 11, these systems are translated back using the original variable names. System $S^{*}_{1}$ mirrors the result of separate optimization with no shared causes. However, three other possibilities exist, all of which share the MOPI $mys\cdot roca$ ($p_6$ in Table 5).

**Table 6:** Multi-output solution to MOPI chart in Table 5

Systems	$S^{*}_{1}$	$S^{*}_{2}$	$S^{*}_{3}$	$S^{*}_{4}$
Output				
*mvta*	$p_{3}$	$p_{3}p_{6}$	$p_{4}p_{6}$	$p_{4}p_{6}$
*ssii*	$p_{10}$	$p_{6}p_{11}$	$p_{6}p_{10}$	$p_{6}p_{11}$

11 $$\begin{aligned} \mathcal {S} = \left\{ \begin{array}{ll} S^{*}_{1} = &{} \left\{ \begin{array}{c} roca \Leftrightarrow mvta\\mys \Leftrightarrow ssii\\ \end{array}\right. \\ S^{*}_{2} = &{} \left\{ \begin{array}{c} mys\cdot roca + roca \Leftrightarrow mvta\\ mys\cdot roca + roca' \Leftrightarrow ssii\\ \end{array}\right. \\ S^{*}_{3} = &{} \left\{ \begin{array}{c} mys\cdot roca + mys' \Leftrightarrow mvta\\ mys\cdot roca + mys \Leftrightarrow ssii\\ \end{array}\right. \\ S^{*}_{4} = &{} \left\{ \begin{array}{c} mys\cdot roca + mys' \Leftrightarrow mvta\\ mys\cdot roca + roca' \Leftrightarrow ssii\\ \end{array}\right. \end{array}\right. \end{aligned}$$

Comparing the PI chart derived from separate optimization in Table 4 with the PI chart derived from multi-output optimization in Table 5, three observations can be made. First, while simple single-output optimization suggests that the two analyzed outputs have nothing in common, multi-output optimization reveals a shared complex cause that feeds into three alternative explanations for the analyzed data. Second, multi-output optimization leads to the identification of a (multi-output) PI that is not part of any PI chart under separate optimization. Third, three PIs (*p*4, $p_{10}$ and $p_{11}$) are present in the PI charts of both separate and joint optimization. However, under separate optimization these PIs are useless because they are dominated by essential PIs, whereas the same PIs become useful under multi-output optimization and thus part of CORA’s solution.

## Conclusions

Modern CCMs, such as QCA and CNA, have started to make inroads into medical and health research over the last decade. At the same time, these methods remain unable to process data on multi-morbidity because such data require the capability to analyze complex effects. In this article, we have presented CORA, a new member of the family of CCMs with which multiple conditions and their complex conjunctions can be analyzed.

CORA takes its inspiration from electrical engineering, and switching circuit analysis in particular. Leveraging this source of inspiration has allowed redundancies, which have prevented a causal interpretation so far when analyzing multiple effects, to be straightforwardly absorbed into CORA’s more general framework. To demonstrate CORA, we have provided several example applications, both with simulated and empirical data, in which CORA has been shown to be able to simultaneously explain individual conditions as well as complex conjunctions of conditions. Through CORA, problems of multi-morbidity in particular, and configurational analyses of complex effects in general, thus come into the analytical reach of CCMs.

Despite the significant advances offered by CORA, important avenues for further refinements in configurational data analysis remain to be explored in future work. For example, researchers currently still have to be able to determine in advance which of the variables in their data belong to the input and which to the output side. A fully naive yet completely open approach to configurational data analysis would let the method determine the assignment. With respect to the more general structure of configurational cause-effect relations, sequential circuit analysis offers yet another possibility to expand the limits of configurational data analysis in significant ways. Incorporating a dimension of sequence would allow analysts to specify the exact order of occurrence of inputs. A third research avenue is provided by heuristic procedures for contexts of (very) big data. These procedures seek to strike a balance between the desire to optimize Boolean functions and the requirement to process high-dimensional data. Lastly, switching circuit theory distinguishes between the analysis of deterministic circuits and that of probabilistic circuits. So far, CCMs have exclusively moved within the realms of the former. A shift towards probabilistic circuits, however, would call for an accompanying change in the theory of causation which CCMs currently work under. The implications of such a move would have to be examined thoroughly.

As varied as the possibilities for advancement are, as large are the challenges and questions to be addressed. Nonetheless, we believe that CORA has demonstrated unequivocally that configurational analysts need not reinvent the wheel. With the famous Quine-McCluskey algorithm, electrical engineering and logic design have developed already in the 1950s the procedures that continue to represent the technical state-of-the-art of QCA. Since the 1950s, however, electrical engineering and logic design have progressed considerably. These fields thus still hold numerous tools on offer that could help researchers to improve their understanding of complex medical and health conditions.

## Supplementary Information

**Additional file 1.**

## Data Availability

Appendices and example datasets used in the section Results are available from https://osf.io/qyb9a/.

## Abbreviations

ASF: Atomic solution formula
CCMs: Configurational Comparative Methods
CNA: Coincidence Analysis
CORA: Combinational Regularity Analysis
CSF: Complex solution formula
MOPI: Multi-output prime implicant
PI: Prime implicant
QCA: Qualitative Comparative Analysis
QMC: Quine-McCluskey algorithm
