# Supplementary material for: Combinational Regularity Analysis (CORA) — a new method for uncovering complex causation in medical and health research
Source: BMC Med Res Methodol. 2022 Dec 23;22:333. doi: 10.1186/s12874-022-01800-9 (PMC9784266; doi:10.1186/s12874-022-01800-9)
Supplement: Supplementary file 1 — Additional file 1. [file 12874_2022_1800_MOESM1_ESM.pdf]

Combinational Regularity Analysis (CORA) — A New Method for Uncovering Complex Causation in Medical and Health Research

Appendix

December 2, 2022

The original causal-chain CSF, seemingly without redundancies, is given in Expression (1), the obviously redundancy-affected equivalent common-cause CSF in Expression (2):

$$(l't' + s \Leftrightarrow x)\,(t + x \Leftrightarrow m)\,, \tag{1}$$

$$(l't' + s \Leftrightarrow x)\,(l't' + s + t \Leftrightarrow m)\,. \tag{2}$$

Since the equivalence operator is a non-fundamental operator, we can replace it and rewrite Expression (1) as Expression (3) and Expression (2) as Expression (4) using only fundamental operators:

$$\Big((l't' + s)' + x\Big)\,(l't' + s + x')\,\big((t + x)' + m\big)\,(t + x + m')\,, \tag{3}$$

$$\Big((l't' + s)' + x\Big)\,(l't' + s + x')\,\Big((l't' + s + t)' + m\Big)\,(l't' + s + t + m')\,. \tag{4}$$

The first two main conjuncts of Expressions (3) and (4), which are identical, can then be Boolean-multiplied out as shown in Expressions (5a) to (5e):

$$\Big((l't'+s)'+x\Big)(l't'+s+x') = \Big((l't')'\,s'+x\Big)(l't'+s+x') \quad \text{by De Morgan,} \tag{5a}$$

$$= \Big(\Big((l')'+(t')\Big)\,s'+x\Big)(l't'+s+x') \quad \text{by De Morgan,} \tag{5b}$$

$$= ((l+t)\,s'+x)(l't'+s+x') \quad \text{by involution,} \tag{5c}$$

$$= (ls'+ts'+x)(l't'+s+x') \quad \text{by distribution,} \tag{5d}$$

$$= ls'x'+ts'x'+l't'x+sx \quad \text{by distribution.} \tag{5e}$$

The last two main conjuncts of Expression (3) can be Boolean-multiplied out as shown in Expressions (6a) to (6b):

$$\big((t+x)'+m\big)(t+x+m') = (t'x'+m)(t+x+m') \quad \text{by De Morgan} \tag{6a}$$

$$= t'x'm'+mt+mx \quad \text{by distribution} \tag{6b}$$

Next, we re-multiply Expression (6b) with Expression (5e) to obtain the fully reduced fundamental form of Expression (1). This process is shown in Expressions (7a) to (7d):

$$(t'x'm'+mt+mx)(ls'x'+ts'x'+l't'x+sx) = ls'x't'm'+ls'x'mt+ts'x'm+l't'xm+sxmt+sxm \quad \text{by distribution,} \tag{7a}$$

$$= ls'x't'm'+ts'x'm\,(t+1)+l't'xm+sxm\,(t+1) \quad \text{by distribution,} \tag{7b}$$

$$= ls'x't'm'+ts'x'm\,(1)+l't'xm+sxm\,(1) \quad \text{by domination} \tag{7c}$$

$$= ls'x't'm'+ts'x'm+l't'xm+sxm \quad \text{by identity} \tag{7d}$$

Subsequently, we proceed the same way with respect to the last two main conjuncts of Expression (4) as shown in Expressions (8a) to (8e):

$$\begin{aligned}
\Big((l't'+s+t)'+m\Big)(l't'+s+t+m') &= \Big((l't')'\,s't'+m\Big)(l'+s+t+m') && \text{by De Morgan,} \quad (8a)\\
&= \Big(\Big((l')'+(t')'\Big)\,s't'+m\Big)(l'+s+t+m') && \text{by De Morgan,} \quad (8b)\\
&= ((l+t)\,s't'+m)(l'+s+t+m') && \text{by involution,} \quad (8c)\\
&= (ls't'+m)(l'+s+t+m') && \text{by distribution,} \quad (8d)\\
&= ls't'm'+ml'+ms+mt && \text{by distribution.} \quad (8e)
\end{aligned}$$

Finally, we re-multiply Expression (8e) with Expression (5e) to obtain the fully reduced fundamental form of Expression (2). This process is shown in Expressions (9a) to (9d):

$$\begin{aligned}
&(ls't'm'+ml'+ms+mt)(ls'x'+ts'x'+l't'x+sx)\\
&= ls'x't'm'+ls'x'mt+ts'x'ml'+ts'x'm+l't'xm+l't'xms+sxml'+sxm+sxmt && \text{by distribution,} \quad (9a)\\
&= ls'x't'm'+ts'x'm\,(l+l')+l't'xm\,(1+s)+sxm\,(1+l'+t) && \text{by distribution,} \quad (9b)\\
&= ls'x't'm'+ts'x'm\,(1)+l't'xm\,(1)+sxm\,(1) && \text{by complementarity and domination,} \quad (9c)\\
&= ls'x't'm'+ts'x'm+l't'xm+sxm && \text{by identity.} \quad (9d)
\end{aligned}$$

Now, it is easy to see that Expression (7d) and Expression (9d) are identical.
